# A Retrospective Cohort Study of the Utility of Ultrasound, 99mTc-Sestamibi Scintigraphy, and Four-Dimensional Computed Tomography for Pre-Operative Localization of Parathyroid Disease To Facilitate Minimally Invasive Parathyroidectomy

**DOI:** 10.7759/cureus.21177

**Published:** 2022-01-12

**Authors:** Christopher R Hillyar, Hirah Rizki, Ruzi Begum, Amit Gupta, Nagesh Nagabhushan, Peng H Lee, Simon Smith

**Affiliations:** 1 Internal Medicine, John Radcliffe Hospital, Oxford University Hospitals NHS Foundation Trust, Oxford, GBR; 2 Surgery, Chelmsford Breast Unit, Broomfield Hospital, Mid and South Essex NHS Foundation Trust, Broomfield, GBR; 3 Radiology, Broomfield Hospital, Mid and South Essex NHS Foundation Trust, Chelmsford, GBR; 4 General Surgery, Broomfield Hospital, Mid and South Essex NHS Foundation Trust, Chelmsford, GBR; 5 Surgery, Chelmsford Breast Unit, Broomfield Hospital, Mid and South Essex NHS Foundation, Broomfield, GBR

**Keywords:** parathyroid disease, localisation, primary hyperparathyroidism, four-dimensional computed tomography, 99m-tc sestamibi scintigraphy, ultrasound (u/s), minimally invasive parathyroidectomy

## Abstract

Background

This study investigated the utility of ultrasound (US), 99mTc-Sestamibi scintigraphy (Sestamibi), and four-dimensional computed tomography (4DCT) for pre-operative localization of a single abnormal parathyroid gland prior to minimally invasive parathyroidectomy (MIP) to determine the optimum pre-operative scans to facilitate a MIP.

Methods

Patients with primary hyperparathyroidism who underwent curative parathyroidectomy at Broomfield Hospital, Mid and South Essex NHS Foundation Trust between 2009 and 2018 were included. Diagnostic performance parameters and the agreement between US, Sestamibi, and 4DCT were evaluated. Cohen’s κ was used to assess the strength of agreement between imaging modalities.

Results

At localizing pathology to the correct side of the neck, Sestamibi had the highest sensitivity (87%), followed by US (76%) and 4DCT (64%). 4DCT had a positive predictive value (PPV) of 95%, similar to Sestamibi (96%), but higher than US (92%). Amongst patients who underwent both US and Sestamibi, the abnormal parathyroid gland was localized to the same area by both imaging modalities in 77% of patients (Cohen’s κ: 0.383). Following an inconclusive US or Sestamibi scan, or discordance between the two modalities, 4DCT was correct at localization in 63% of patients.

Conclusion

Sestamibi has the highest sensitivity and PPV for accurately localizing parathyroid pathology. The addition of US to a positive Sestamibi scan adds little additional value. 4DCT is the preferred imaging modality following an inconclusive Sestamibi or US.

## Introduction

Primary hyperparathyroidism (PHPT) is an endocrine disorder characterized by increased release of calcium from skeletal hydroxyapatite, leading to hypercalcemia due to an inappropriately normal or elevated plasma parathyroid hormone (PTH) level. In the 1970s, with the advent of multichannel biochemical screening, the incidence of PHPT rose sharply [1]. Since then, there has been a steady worldwide rise in its prevalence [2].

PHPT is predominantly caused by a solitary benign adenoma [3-6]. Although most patients with PHPT are largely asymptomatic, and diagnosis may be made incidentally on routine blood tests, epidemiological studies have demonstrated that if untreated, PHPT inevitably results in gastrointestinal dysfunction, impaired renal function and nephrolithiasis, reduced bone mineral density and fragility fractures, and/or psychiatric disturbances [7,8]. The American Association of Endocrine Surgeons guidelines recommends that parathyroidectomy be offered to all symptomatic patients with PHPT. For asymptomatic PHPT patients, parathyroidectomy may also be considered a therapeutic option [9].

Historically, a bilateral neck exploration and excision of a macroscopically abnormal parathyroid gland or glands were considered the gold standard operation [10]. In the 21st century, however, there has been a move towards more focused approaches, with several randomized controlled trials investigating the merits and pitfalls of a unilateral neck exploration [11-13]. In 2002, Bergenfelz et al. conducted the first unselected randomized controlled trial of unilateral neck exploration for PHPT. This study identified a higher incidence of early severe symptomatic hypocalcemia following a bilateral neck exploration than patients who received unilateral neck exploration [11]. Since then, further randomized trials have added to the body of evidence supporting a unilateral neck exploration by demonstrating shorter operative times and reduced morbidity as some of the key benefits [12,13]. Alternative approaches have also been reported, with Miccoli et al. describing their experience with a video-assisted parathyroidectomy [14] and Henry et al. publishing their results from an endoscopic approach [15].

More commonly, however, surgeons adopt open, minimally invasive parathyroidectomy (MIP) in patients with a radiologically proven single enlarged parathyroid adenoma. In Kunstman et al., this approach was associated with numerous secondary benefits, including decreased hospital cost, improved patient satisfaction, decreased operative time, and same-day discharge [3]. According to Kluijfhout et al., the key to undertaking MIP is the pre-operative localization of the pathological parathyroid gland, ideally to a specific quadrant in the neck [16]. There are many scans available to facilitate localization, including ultrasound (US), 99mTc-sestamibi scintigraphy (Sestamibi), four-dimensional computed tomography (4DCT), and magnetic resonance imaging (MRI). These imaging modalities can be utilized alone or in combination, each with advantages and disadvantages. Neck US is often a first-line imaging modality utilized in most centers as it is inexpensive, non-invasive, and often reproducible on the operating table. In addition, the US allows for thyroid nodules to be biopsied at the time of the scan if required. However, the US is operator-dependent and limited by a high body mass index (BMI) and reduced neck extension.

In the United Kingdom (UK), there are currently no standardized investigations or referral criteria to guide decision-making regarding PHPT identified in primary care. There is variability in the utilization of different diagnostic tests and imaging modes in secondary care. The UK National Institute of Health and Care Excellent (NICE) recommends using one imaging modality prior to surgery and the second form of imaging only if this is thought to add value [17].

The main objective of this study was to identify the optimal pre-operative scan, or combination of scans, to accurately localize a single pathologically abnormal parathyroid gland to a specified side or quadrant in the neck, thereby facilitating a MIP approach. This objective was met by evaluating the diagnostic performance and interobserver agreement between US, Sestamibi, and 4DCT imaging. A comparison was made between the location of the abnormal parathyroid gland as per the diagnostic imaging reports and the location of the abnormal gland as identified during surgery. This article was previously presented as a meeting abstract at the 2020 European Society of Radiology on July 15th-19th, 2020.

## Materials and methods

Patient population and data collection

A retrospective analysis of electronic patient records (EPR) was undertaken at a single secondary care unit at Broomfield Hospital, Mid and South Essex NHS Foundation Trust, UK. All patients treated surgically with a parathyroidectomy over 10 years (January 1st, 2009 to December 31st, 2018) were eligible for consideration for inclusion in this study. This included individuals who met the UK NICE guidelines for PHPT requiring surgical intervention [17]. Specifically, these were patients with symptoms of hypercalcemia, such as thirst, frequent or excessive urination, or constipation, end-organ disease (renal stones, fragility fractures, or osteoporosis), or an albumin-adjusted serum calcium level of ≥2.85 mmol/L. Prior to surgery, all patients underwent an assessment of their vitamin D levels. Familial hypocalciuric hypercalcemia was assessed and excluded on a case by cases basis.

The final cohort of included patients included those with a successful, curative, surgical excision of a single pathologically abnormal parathyroid gland and pre-operative elevated serum calcium above normal levels as defined by NICE guidelines, followed by a sustained return to normocalcaemia in the post-operative period. All included patients also had a post-operative histologically proven single gland disease, defined as identifying a macroscopically abnormal parathyroid gland at the time of surgery with a subsequent diagnosis of a single parathyroid adenoma, hyperplasia, oxyphil, or carcinoma on final histology. Patients with histological multi-gland pathology or recurrent PHPT requiring further surgery were excluded. Patients with incomplete or missing surgical, histological or radiological data were also excluded.

Data extracted from EPR included diagnostic, operative, histological, and outcomes data. Radiology reports for each patient were obtained from the Picture and Archiving Communication Systems (PACS).

Operative approach

The location of the pathological parathyroid intra-operatively (as described in the operation note) to a side or a quadrant in the neck was considered the gold standard for comparison with imaging. The parathyroid operations employed either a midline collar incision, where some doubt remained as to the exact side of the pathological gland or a MIP with a lateral approach, where the parathyroid was presumed well-localized pre-operative imaging. Intraoperative calcium and biochemical analysis of parathyroid hormone levels were used case-by-case for any diagnostic uncertainty.

Imaging

Ultrasound

A group of radiologists performed ultrasound and specialist ultra-sonographers trained in parathyroid adenoma detection. Imaging was performed in neck extended position with a high-frequency linear transducer (8 to 15MHz) depending on neck size and patient habitus.

Sestamibi

A dual-phase 99mTc-sestamibi washout study was performed as early phase 20-minute and late phase 120-minute planar images. Planar images were obtained on a 256 x 256 matrix at 140keV 15% to 20% window. Two cameras were used. Single-photon emission computer tomograms (SPECT) were performed at 150 minutes in most patients, particularly when the planar images did not demonstrate an apparent nodule or washout was suboptimal on the 120-minute images. SPECT was obtained on a 128 x 128 matrix, zoom 1.46, at 140keV 15% or 20% window, with 64 projections with a total scan time of 5 minutes.

Four-Dimensional Computed Tomography

4DCT studies were performed on a Toshiba Aquillion CX 64 detector scanner with a rotation time of 500ms, the pitch of 64x, tube voltage of 120kVP, and smart mA acquisition (nQ mA algorithm, Toshiba), as a multi-phasic technique with coverage from internal auditory meatus down to the mid-sternum. After a precontrast acquisition, images were obtained at 25 and 80 seconds following contrast as a “triple phase study.” The weight-based volume of Ioversol (Optiray 350) was used as contrast media and given at a rate of 3ml/s through a pump injector.

Statistical Analysis

The diagnostic performance and interobserver agreement between imaging modalities were assessed. Where histological analysis concluded the presence of a single pathological parathyroid gland, the operative site was considered the gold standard for comparison to an imaging location. The sensitivity/true positive rate (TPR), false-negative rate (FNR), positive predictive value (PPV), and false detection rate (FDR) of each imaging modality at correctly localizing parathyroid disease to the correct side or quadrant of the neck, was calculated. Cohen’s κ was used to determine concordance between imaging modalities. To assess the concordance between imaging modalities, the anatomical location identified for each test was categorized into one of either left, left upper, left lower, right, right upper, right lower, ectopic, or not localized (inconclusive). Calculations were based on true positive scans that localized the pathological parathyroid gland to the correct side/quadrant in the neck as found at surgery. A false positive scan identified a pathological gland but localized it to the incorrect side/quadrant in the neck. Inconclusive scans, where no abnormal parathyroid gland was seen on imaging, were treated as false negatives. There were no true negative scans as all patients included in this study had a single pathological parathyroid gland excised at the surgery. Table 1 summarises the test positive and negative test cases. Data handling was conducted using Microsoft Excel 2013. Statistical analysis was performed with GraphPad Prism 9.2.0.

**Table 1:** Summary of test positive and test negative cases for the purposes of statistical analysis

	Correct	Incorrect
Scan location = location at surgery	True positive	
Scan location ≠ location at surgery		False-positive
No pathological parathyroid gland was seen on imaging, but an abnormal gland was found at surgery		Inconclusive

## Results

Patient characteristics

This study reviewed 203 consecutive patients treated for PHPT. Forty-seven patients did not meet the inclusion criteria, 156 patients were included in the final analysis. These patients had a mean age of 62 years and 11 months (SD 12 years and one month) at the time of surgery.

On final histology, a diagnosis of parathyroid adenoma was obtained in 87% of patients (n=136). The operation note specified the side of the neck-bearing disease for all 156 included patients, of which 76 had disease located on the left and 80 on the right side of the neck. Localization of parathyroid disease to a quadrant of the neck was specified in the operation note of 140/156 patients. Table 2 summarises these patient characteristics.

**Table 2:** Patient characteristics SD, standard deviation

n	156 patients
Age, mean (SD)	62 years 11 months (12 years 1 month)
Histology, n (%)	
Parathyroid adenoma	136 (87%)
Parathyroid hyperplasia	19 (12%)
Parathyroid carcinoma	1 (0.006%)
Location at surgery, n (%)	
Side	Specified in n=156
Right	80 (51%)
Left	76 (49%)
Quadrant	Specified in n=140
Right upper	29 (21%)
Right lower	40 (29%)
Left upper	34 (24%)
Left lower	37 (26%)

Imaging

**Figure 1:**
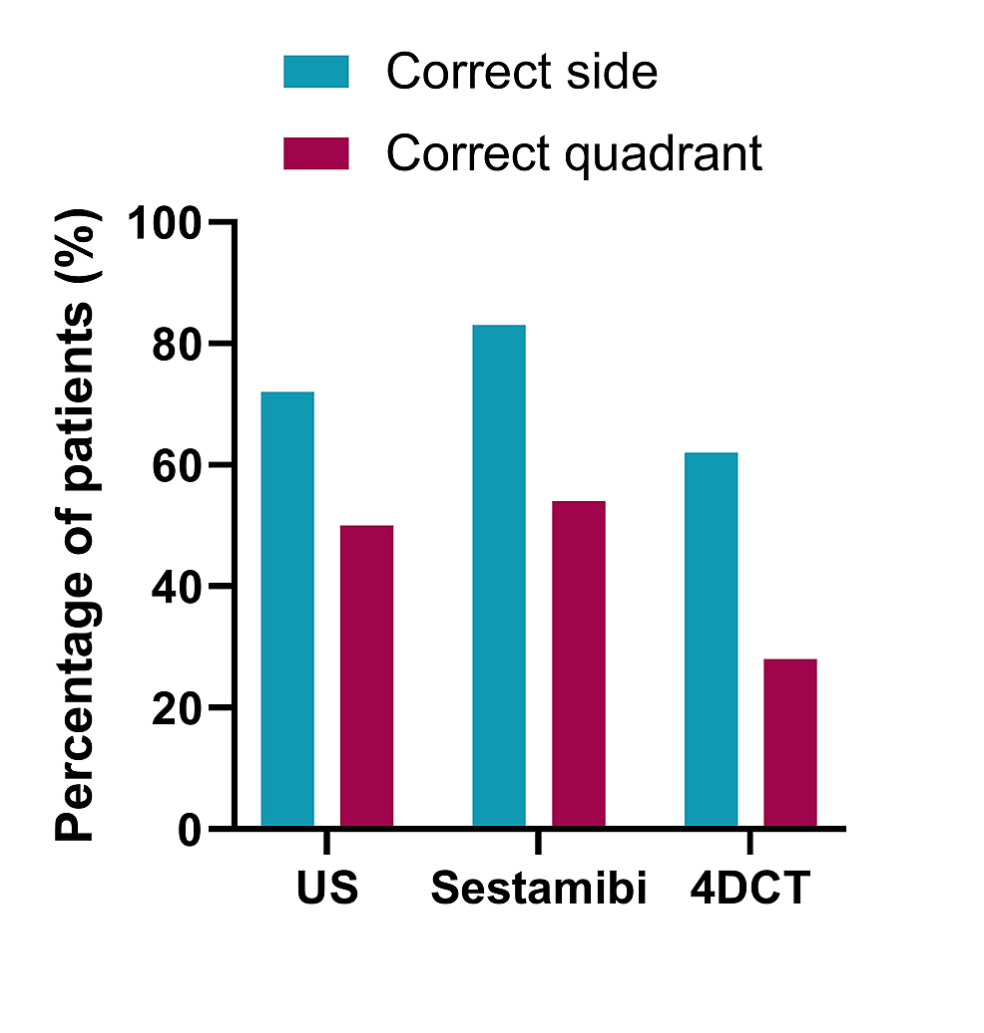
Percentage of patients with disease localized to the correct side or the correct quadrant of neck Percentage of patients with a true positive scan shown. US, ultrasound scan; Sestamibi, 99mTc-sestamibi scintigraphy; 4DCT, four-dimensional computed tomography scan.

In total, 152 US, 155 Sestamibi, and 29 4DCT scans were performed. Most patients were investigated pre-operatively using two different imaging modalities (n=123). Only four patients had a pre-operative scan, and 29 received all three scans. Figure 1 summarises the utilization of pre-operative imaging in our patient cohort.

Ultrasound

The US correctly identified the presence of a pathological parathyroid gland in the neck in 118/152 patients (78%). The US was inconclusive (i.e., failed to visualize an abnormal parathyroid gland anywhere in the neck) in 34 patients (22%). All patients with the inconclusive US had a Sestamibi scan, which correctly identified the presence of a pathological gland in 23/34 patients (68%) and remained inconclusive for 11/34 patients (32%). Twenty-two patients with an inconclusive US scan also underwent a subsequent 4DCT scan which correctly identified the presence of a pathological parathyroid gland in the neck for 14/22 patients (64%) but remained inconclusive for 8/22 patients (36%).

Sestamibi

Sestamibi correctly identified the presence of a pathological parathyroid gland in the neck in 129/155 patients (83%). It failed to identify any abnormal parathyroid gland in the neck of 20/155 patients scanned (13%). Of these 20 patients, 4DCT and the US correctly identified the presence of a pathological gland in the neck in 10 (50%) and nine (45%) patients, respectively. For the remaining patient, both scans remained inconclusive. 

Four-Dimensional Computed Tomography

4DCT correctly identified the presence of a pathological parathyroid gland in the neck in 19/29 patients (66%). The remaining scans were inconclusive and failed to report abnormal parathyroid glands in the neck. Of the 29 4DCT scans, 19 were performed in patients in whom the Sestamibi and US findings were discordant. A subsequent 4DCT scan correctly located the pathological gland in the neck in 12/19 (63%) patients.

Four patients had no parathyroid pathology identified by all three imaging modalities. Of these four patients, three (75%) had a parathyroid adenoma, and one (25%) had parathyroid hyperplasia affecting a single gland identified at surgery.

Localization of parathyroid disease to the correct side of the neck

**Figure 2:**
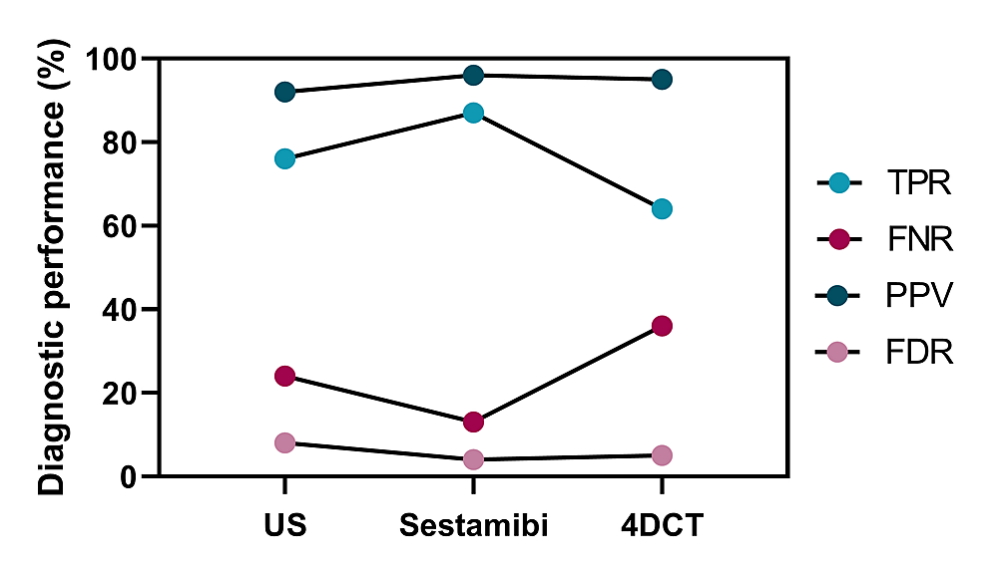
True positive rate, false-negative rate, positive predictive value, and false discovery rate for US, Sestamibi, and 4DCT at localizing parathyroid disease to the correct side of the neck Diagnostic performance parameters are shown for each scan type. TPR, true positive rate; FNR, false-negative rate; PPV, positive predictive value; FDR, false discovery rate; 4DCT, Four-Dimensional Computed Tomography.

Regarding localization of parathyroid disease to the correct side of the neck, Sestamibi had the highest TPR (87%), followed by US (76%). The TPR of 4DCT was the poorest of the three imaging modalities (64%). The FNR of Sestamibi was the lowest (13%), followed by the US (24%). The FNR of 4DCT was the poorest of the three imaging modalities (36%). The PPV - that is, where a pathological gland was identified, its location was attributed to the correct side of the neck - was similar for all three imaging modalities. The PPV for Sestamibi, 4DCT, and US was 96%, 95%, and 92%, respectively. The FDR was also similar for all three imaging modalities, with Sestamibi, 4DCT, and US having an FDR of 4%, 5%, and 8%, respectively (Figure 2).

**Figure 3:**
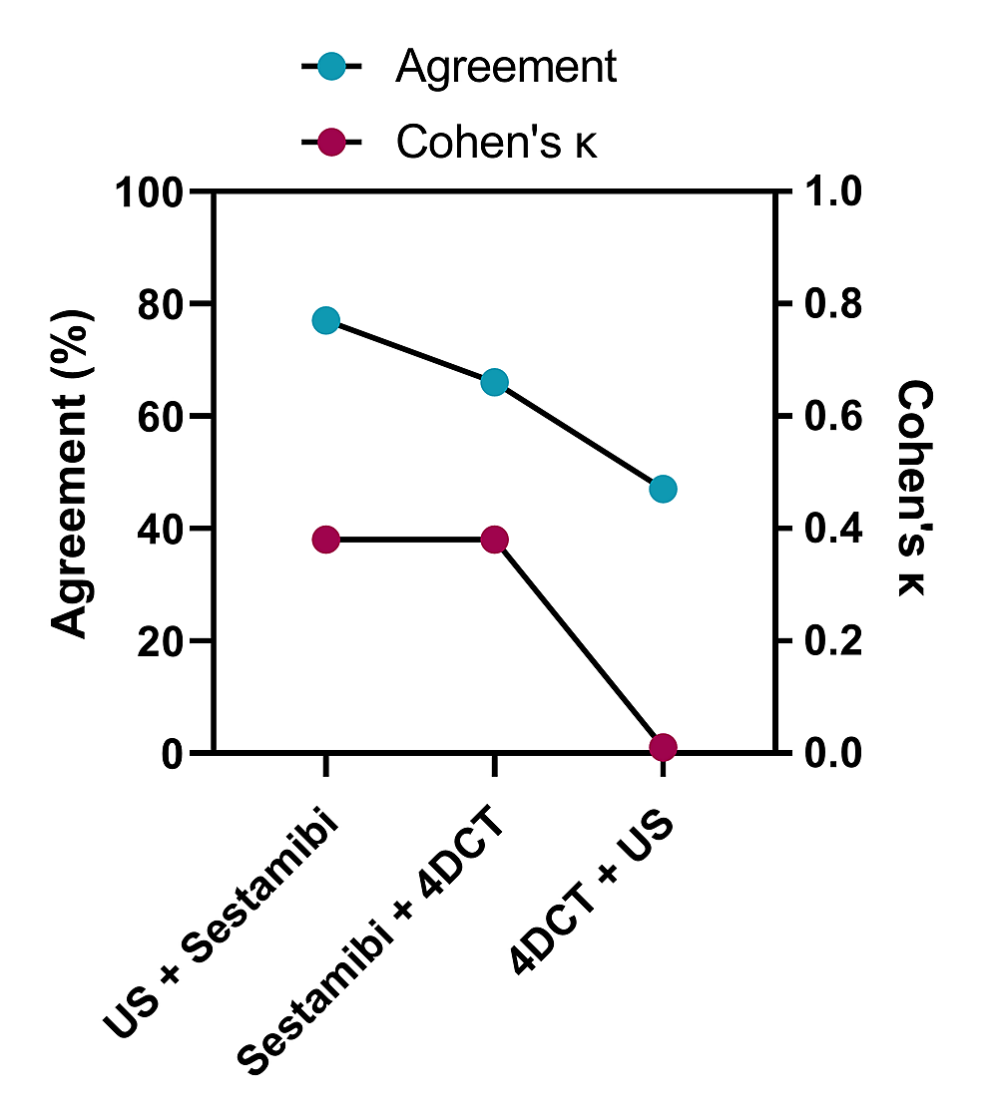
Percentage agreement, and Cohen’s kappa between the US, Sestamibi, and 4DCT at localizing parathyroid disease to the correct side of the neck Scan report agreement (percentage of patients) and Cohen’s κ for each combination of two scan types; US, ultrasound scan; Sestamibi, 99mTc-sestamibi scintigraphy; 4DCT, four-dimensional computed tomography scan

Cohen’s κ analysis was utilized to assess the strength of agreement between imaging modalities in terms of their ability to localize parathyroid disease to the side of the neck. For Sestamibi, Cohen’s κ indicated the addition of US in the same patient was likely to identify the pathological gland in the exact location or remain inconclusive due to a location agreement seen between Sestamibi and US in 77% of patients (Cohen’s κ: 0.38). The addition of 4DCT to Sestamibi yielded the same result in 66% of patients (Cohen’s κ: 0.38). By contrast, the scan agreement between US and 4DCT was only 47% (Cohen’s κ: 0.01) (Figure 3).

Localization of parathyroid disease to the correct quadrant in the neck

A sub-analysis, which included 140 patients, was performed to assess the ability of US, Sestamibi, and 4DCT’s ability to localize parathyroid disease to the correct quadrant in the neck. These patients had a mean age of 62 years and 11 months (SD 12 years and five months) at the time of surgery. After exclusion of imaging reports which did not specify a quadrant in the neck, a total of 133 US, 135 Sestamibi, and 25 4DCT scans were included in the sub-analysis.

**Figure 4:**
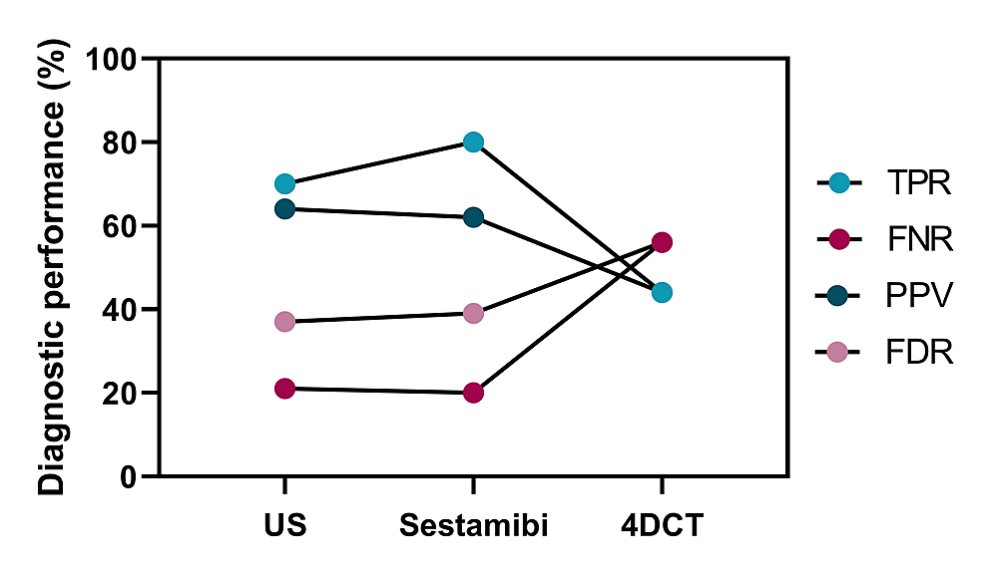
True positive rate, false-negative rate, positive predictive value, and false discovery rate for US, Sestamibi, and 4DCT at localizing parathyroid disease to the correct quadrant of the neck Diagnostic performance parameters are shown for each scan type. TPR, true positive rate; FNR, false negative rate; PPV, positive predictive value; FDR, false discovery rate.

In terms of localizing parathyroid disease to the correct quadrant in the neck, Sestamibi had the highest TPR (80%), followed by US (70%). The sensitivity of 4DCT was the poorest of the three imaging modalities (44%). The FNR of Sestamibi and the US were similar (20% and 21%, respectively), but the FNR of 4DCT was much poorer (56%). The PPV of Sestamibi and the US were also similar (62% and 64%, respectively), while 4DCT was much poorer (44%). Finally, the FDR was also similar for US and Sestamibi (37% and 39%, respectively), but 4DCT had a much poorer FDR (56%) (Figure 4).

**Figure 5:**
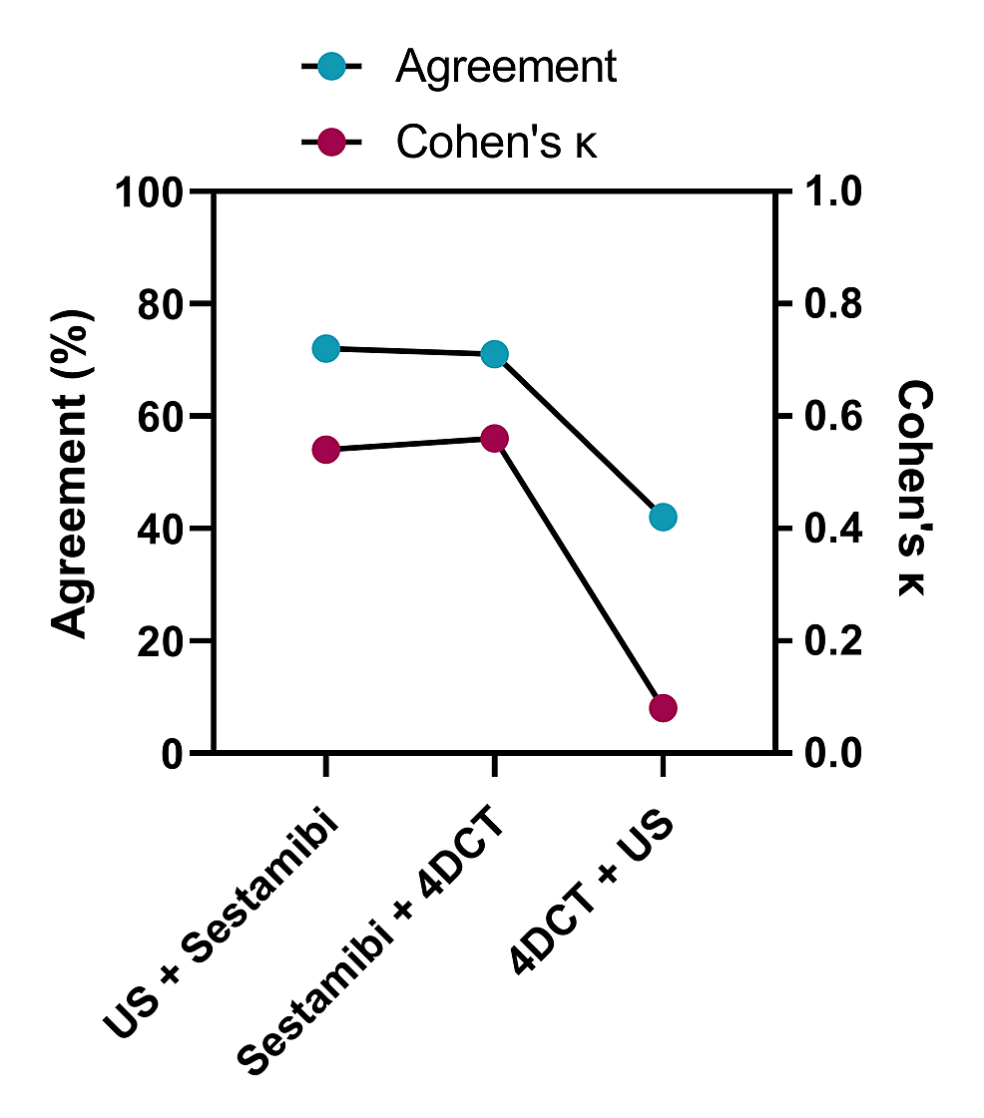
Percentage agreement and Cohen’s kappa between US, Sestamibi, and 4DCT at localizing parathyroid disease to the correct quadrant of the neck Scan report agreement (percentage of patients) and Cohen’s κ for each combination of two scan types; US, ultrasound scan; Sestamibi, 99mTc-sestamibi scintigraphy; 4DCT, four-dimensional computed tomography scan

Cohen’s κ was also utilized to assess the strength of the agreement between imaging modalities in terms of the ability to localize parathyroid disease to the correct quadrant in the neck. For Sestamibi, Cohen’s κ indicated that the addition of US in the same patient was likely to yield a similar result, with quadrant location agreement between Sestamibi and US scans of 72% (Cohen’s κ: 0.54). The addition of the 4DCT scan to the Sestamibi scan was 71% likely to yield a similar result (Cohen’s κ: 0.56). By contrast, the agreement between US and 4DCT was only 42% (Cohen’s κ: 0.08) (Figure 5).

## Discussion

Although there are no clear UK guidelines regarding which patients with PHPT should be operated upon, the American Association of Endocrine Surgeons Guidelines stipulates that surgery should be offered to symptomatic patients with PHPT [9]. Asymptomatic patients also benefit from surgery to prevent the known sequelae of PHPT [18]. In addition, it is argued that no patient is truly asymptomatic and may indeed be suffering from vague neuro-cognitive symptoms, which are seen to improve significantly after treatment [18]. However, taking a well, asymptomatic patient and exposing them to the risks of a parathyroidectomy needs to be carefully weighed. This is where the MIP approach is most relevant. 

Randomized trials comparing the focused MIP with the traditional bilateral approach have shown similar cure and recurrence rates [4,11,13,19]. A recent systematic review and meta-analysis have shown that MIP has a shorter mean operative time (103 versus 64 minutes) and lower overall complication rates (17% versus 4%) compared with the traditional bilateral approach [19]. Other studies have shown similar advantages to adopting a MIP approach with patients reporting lower post-operative pain, lower analgesic requirements, shorter scar length, and improved patient satisfaction [3,13]. For these reasons, we advocate for a MIP approach where possible, and underpinning a successful MIP is the accurate pre-operative localization of the pathological parathyroid gland [5].

In the absence of UK guidelines, this study aimed to determine the optimum scan, or combination of scans, to facilitate a MIP through accurate pre-operative localization. Although previously published data have focused on the correct identification of the diseased parathyroid gland only, we build on this by looking for the correct identification of parathyroid disease, not only to a side of the neck but more specifically to a quadrant within the neck. A MIP is not routinely recommended for known or suspected multi-gland disease [20]. Therefore this study excluded patients with multiple pathological glands found intra-operatively.

Of the three localization scans used in our Trust, our study has identified Sestimibi as having the highest sensitivity/TPR at correctly identifying the laterality and quadrant of the pathological parathyroid gland in the neck. This is in keeping with a recent meta-analysis that determined a pooled sensitivity for Sestamibi of 83% [21].

The most common approach to localization in a patient with a de novo diagnosis of PHPT is combining Sestamibi with the US, with 62% of surgeons in the United States reporting this as their preferred approach [22]. However, in the UK, NICE recommends using one imaging modality prior to surgery and the second form of imaging only if this will add value [17]. In our study, 97% of patients had at least Sestamibi and US scans, with a ‘fair’ degree of concordance in localization between the two imaging modalities. This implies that the addition of US after a positive Sestamibi scan does not add value as it is likely to reproduce the same findings. This is supported by Smith et al., who demonstrated localization of parathyroid disease by the US was accurate in 82% of patients. At the same time, the accuracy of Sestamibi was similar, albeit slightly higher (85%) [23]. In addition, a meta-analysis conducted in 2017 reported no significant difference between the US and Sestamibi in terms of sensitivity and specificity [21]. Due to the agreement between US and Sestamibi, Aarum et al. reported that MIP is not cost-effective if concordant results of Sestamibi and ultrasonography are obtained prior to conducting a parathyroid operation [24]. Thus, it is argued that Sestamibi is only indicated when the US produces inconclusive results [25].

However, a systematic review and meta-analysis by Carral et al. reported that the sensitivity of US in locating a diseased parathyroid gland ranges from 76% to 91% [26]. This large range may be attributed to many factors. Firstly, the US is highly operator-dependent [25]. Secondly, the US may be affected by patient factors, such as obesity and the inability to extend their neck adequately. A third limitation is that the diagnosis of parathyroid disease is affected by adjacent thyroid nodules, goiters, the position of the diseased parathyroid gland, and its size [21]. The sensitivity of US is reduced in detecting smaller pathological glands, falling by 15% in glands under 1 cm (from 85% to 70%) [26]. The United States guidelines recommend utilizing Sestamibi and US because this has increased sensitivity [9]. In contrast, the UK NICE guidelines advocate undertaking additional imaging only to add value [17]. The results of this study support the UK NICE guidelines in that where a Sestamibi scan identifies an abnormal parathyroid gland, the addition of a US may not add any further value in the majority of patients, because in over two-thirds of individuals in our cohort, US yielded the same results as the Sestamibi scan.

In this study, 4DCT was employed for most patients when the US and Sestamibi findings were discordant. However, 4DCT was found to have the lowest sensitivity for localizing parathyroid disease to the correct side of the neck, and it also had the lowest agreement with US and Sestamibi. This is contrary to published data demonstrating that 4DCT has a similar diagnostic performance to Sestamibi but at the cost of a higher radiation dose [27]. A significant limitation to the interpretation of the results for 4DCT is the small cohort size (only 29 patients), which is likely to render the accuracy of these results more susceptible to random variation in comparison to the results obtained from larger cohorts of patients that underwent US or Sestamibi (152 and 155 patients, respectively).

In addition, there had been a change in the local 4DCT protocol in 2016, the effect of which we do not yet know. The initial protocol consisted of four phases: unenhanced and three-phase contrast image acquisition. Due to the concern regarding the radiation dose, this was altered to unenhanced and two-phase contrast image acquisition. Although the original description of 4DCT by Rodgers et al., local departments have developed modified protocols, there is still debate whether reducing the number of phases could reduce the detection rate [28,29]. Finally, the effect of reader specificity for 4DCT was not analyzed. There are variations in the anatomical location of the parathyroid glands, most notably the inferior and ectopic parathyroid glands. Understanding embryology and the gland’s potential location may differ between dedicated head and neck specialists and general radiologists. However, the collection of information on the proportion of scans read by specialists and general radiologists was outside the scope of this study.

This limitation is also a factor in the subanalysis and the agreement analysis. However, 4DCT was found to have a high PPV similar to Sestamibi. When employed in patients with the inconclusive US, 4DCT identified the gland to the correct side and quadrant in 100% of patients. Following an inconclusive Sestamibi scan, 4DCT correctly localized pathological gland to the correct side of the neck in 50% of patients. Future work focusing on the influence of local protocols and subspeciality background on the reporting of parathyroid scans might adjust outcomes by accounting for these confounding factors.

## Conclusions

The results of this study suggest that Sestamibi, in comparison to US or 4DCT, has higher sensitivity at correctly localizing pathological parathyroid disease to the correct side and quadrant in the neck. When a Sestamibi scan can correctly localize parathyroid disease to a side of the neck, it has a PPV of 96%. Adding the US to a positive Sestamibi scan adds little additional value in the localization of a pathological parathyroid gland in the neck because it is highly likely to yield the same result. Following an inconclusive Sestamibi or US, 4DCT adds the most value for the localization of a pathological parathyroid gland to facilitate a MIP.
